# The effect of hunger on the acoustic individuality in begging calls of a colonially breeding weaver bird

**DOI:** 10.1186/1472-6785-11-3

**Published:** 2011-01-26

**Authors:** Hendrik Reers, Alain Jacot

**Affiliations:** 1 Behavioural Ecology and Evolutionary Genetics Group, Max Planck Institute for Ornithology, Seewiesen, D 82319, Germany; 2 Swiss Ornithological Institute, Field Station Valais, CH-3970 Salgesch, Switzerland

## Abstract

**Background:**

In colonially breeding birds, the ability to discriminate between individuals is often essential. During post-fledging care, parents have to recognize their own offspring among many other unrelated chicks in the breeding colony. It is well known that fledglings and food-provisioning parents of many bird species use contact calls to convey their identity. These calls are also often used as hunger-related signals of need in young birds. Here, we investigate how such calls incorporate signals of need and at the same time act as reliable indicators of each chick's identity.

**Results:**

In a field study, we experimentally manipulated the hunger level of colonially breeding Jackson's golden-backed weaver (*Ploceus jacksoni*) nestlings close to fledging and investigated its effects on acoustic call parameters. Some acoustic parameters that were related to the time-frequency pattern showed high individuality and were largely unaffected by a nestling's state of hunger. However, the majority of call parameters were significantly affected by hunger. Interestingly, most of these acoustic parameters showed both consistent changes with hunger and high between-individual differences, i.e. potential for individual recognition.

**Conclusion:**

The results indicate that individual recognition processes can be based on static, hunger-independent call parameters, but also on dynamic hunger-related parameters that show high individuality. Furthermore, these signal properties suggest that the assessment of signals of need can be improved if the signal value is referenced to a chick's vocal spectrum.

## Background

Acoustic signalling in birds is a popular system in which to ask questions about the evolution of identity signalling systems and the costs and benefits of reliably communicating a sender's condition. These two different kinds of signals (i.e. identity and condition) require very different trait properties. Signals of identity need to be relatively consistent over time within individuals to reliably indicate the senders identity [for review see [1]]. In contrast, condition signals, indicating short term changes in condition (e.g. hunger in food-dependent young), need to be plastic within an individual to reliably reflect the different conditions of a sender [e.g. [2,3]]. Both types of signals have been studied separately in parent-offspring communication. However, these two signals occur simultaneously in begging calls of young birds and the question arises: how can one acoustic signal manifest the need for simultaneous high and low intra-individual variation? So far, no study has investigated both types of signals and their contribution to the acoustic variability in a parent-offspring communication system.

In many colonially breeding species, parents need to discriminate their mobile chicks from other conspecific young [4]. In most species, offspring still rely on parental care after a post-fledging phase and the accurate recognition of own offspring is important. Although the crucial time period for parent-offspring recognition is relatively short (i.e. ranging from a few days to several months), one expects selection for reliable recognition mechanism. In species in which misidentification is likely, failure of parents to recognize their offspring is prone to entail fitness costs for both, parents (i.e. reduced reproductive success) and offspring (i.e. starvation). Acoustic parent-offspring recognition has been shown in colonially breeding seabirds [e.g. [5-8]], with a special focus on penguins [e.g. [9]], and in fewer studies on songbirds [e.g. [10,11,8]]. Frequency modulation (FM) has been found to be an important cue for acoustic individual recognition in birds. For example, king penguin (*Aptenodytes patagonicu*s) chicks recognize their parents by FM patterns in their call [12] and zebra finches (*Taeniopygia guttata*) facilitate mate recognition by using FM cues [13]. Although these studies establish that FM is important for acoustic recognition, most likely a combination of different acoustic parameters is used by the receiver to recognise the signaller. By using multiple auditory components, individuals may increase the information content of the call, which is expected to facilitate recognition [4,14].

Many studies have also shown that begging calls incorporate information about a chick's energy requirements or body condition (i.e. signals of need) in an effort to solicit food from parents or compete with siblings [e.g. [15-19]]. These 'need' signals are highly dynamic (i.e. variable with changes in state of need) and exhibit large intra-individual gradual variation; such variation has been correlated to a chick's body condition or hunger level [e.g. [2,20,21]]. An increase in begging intensity is often associated with an increase in call rate, call amplitude and begging bout length [reviewed in [18], but see [22]]. Compared to these characteristics of call delivery, the influence of hunger on acoustic parameters of individual calls has been studied in relatively few species [e.g. [3,21-24]].

Consequently, in species where selection favours individual recognition, begging calls are likely to incorporate both identity cues and signals of need. Therefore, begging calls represent an excellent system to investigate the expression pattern of static [1] and dynamic traits [20] within the same acoustic signal. In a field study with Jackson's golden-backed weavers (*Ploceus jacksoni*) we aim to identify how variation in begging calls simultaneously reflects changes in hunger and incorporates individual distinctiveness. This species is perfectly suited for studying the evolution of individual signatures in begging. It is a colony breeder with highly synchronized breeding at the start of the rainy season. This high synchronization leads to simultaneous fledging on a massive scale, in which parents must be able to locate their offspring in order to provide post-fledging parental care (e.g. food provisioning) [25].

In a first step, we experimentally manipulated hunger levels of nestlings close to fledging and predicted that this treatment would change acoustic parameters in relation to a nestling's hunger level. The time point is important because we predict individual signatures to be developed closely before fledging to enable parents to learn individual signatures while the nestling is still in the nest. Following this, we analyzed the variance components in the begging calls to apportion the observed variance to differences between hunger levels and individuals. Our prediction was that begging calls contain stable individual information over varying hunger levels to reliably indicate a nestling's identity and dynamic information about a chicks hunger. In a last step, we used multivariate methods (i.e. discriminant function analyses) to demonstrate statistically if and how distinctiveness of individuals changes with hunger.

## Results

### Behavioural and acoustic response to food deprivation

Food deprivation affected both begging posture (LMM: b ± SE = 0.99 ± 0.05 posture index/h, p < 0.001, N = 45 individuals) and call rate (LMM: b ± SE = 5.23 ± 0.37 calls in 10 sec/h, p < 0.001, N = 46 individuals). Call rate (calls/10 seconds) was nearly twice as high in hungry chicks (hunger time = 120 min: mean ± SE, 16.7 ± 4.7) compared to satiated chicks (hunger time = 15 min: mean ± SE, 8.6 ± 6.7). Neither the sex of the nestling nor its hatching order had an influence on posture or call rate (all p > 0.24). These results demonstrate that the experiment effectively altered the hunger state of chicks, a prerequisite for investigating the static and dynamic properties of call characteristics important in signalling need and individuality.

Acoustic response to food deprivation was measured for both call parts separately (i.e. first 'whistle-like' part and second 'trill' part). Few call parameters showed clear static call properties, i.e. traits that did not change with hunger level (p > 0.05; Table 1). In the first call part mean frequency modulation and variance in amplitude modulation were not significantly affected by hunger (Table 1). Variance in entropy was not significantly affected by hunger in both call parts (Table 1). Additionally, in the second call part, mean frequency was not significantly affected by hunger (Table 1).

**Table 1:** Effect of hunger on identity in acoustic parameters.

Acoustic parameter			Effect of hunger treatment ^a^			Variance components (in %)			
			Estimate	p-value		Hunger	ID	Nest	Residual
	Duration (ms)		-4.17 ± 1.23	< 0.001		2.2	37.8	20.9	39.2
	Amplitude (dB)	mean	2.98 ± 0.26	< 0.001		11.8	30.4	17.9	39.8
	Amplitude modulation (1/ms)	variance	(-0.02 ± 0.08) x10^-3^	0.753		0.2	21.4	11.0	67.4
	Frequency (Hz)	mean	-239.59 ± 36.87	< 0.001		3.9	31.5	19.2	45.4
Part 1	Frequency modulation	mean	0.03 ± 0.34	0.926		0.4	43.7	20.9	35.0
	Frequency modulation	variance	12.57 ± 5.19	0.016		0.3	35.7	12.7	51.3
	Entropy	(log) mean	0.19 ± 0.03	< 0.001		3.6	22.8	37.4	36.2
	Entropy	variance	(-0.36 ± 10.85) x10^-3^	0.974		0.7	26.3	7.6	65.4
	Pitch (Hz)	mean	-289.59 ± 44.52	< 0.001		3.8	38.1	0.0	58.0
	Pitch goodness	mean	24.33 ± 3.24	< 0.001		5.2	31.6	12.5	50.8
	Duration (ms)		15.85 ± 0.94	**< 0.001**		18.5	50.9	4.6	26.0
	Amplitude (dB)	mean	4.58 ± 0.26	**< 0.001**		24.2	28.8	17.7	29.4
	Amplitude modulation (1/ms)	(log) variance	(-0.27 ± 0.06) x10^-3^	**< 0.001**		2.3	9.5	7.4	80.7
	Frequency (Hz)	mean	-32.08 ± 37.33	0.391		0.3	32.7	7.6	59.4
Part 2	Frequency modulation	mean	1.39 ± 0.32	**< 0.001**		1.1	50.6	9.6	38.7
	Frequency modulation	variance	11.94 ± 6.00	0.047		0.6	18.5	9.2	71.7
	Entropy	(log) mean	0.26 ± 0.03	**< 0.001**		6.2	26.6	21.2	46.0
	Entropy	variance	(-1.43 ± 0.94) x10^-2^	0.128		0.0	31.6	1.4	66.9
	Pitch (Hz)	mean	-135.20 ± 48.15	0.005		0.4	42.0	1.5	56.2
	Pitch goodness	mean	38.60 ± 3.82	**< 0.001**		7.5	17.8	30.0	44.8

The effect of the hunger treatment on acoustic parameters and their variance components according to the differences in time stages of the experimental treatment (Hunger), identity (ID), origin (Nest) and unexplained variance (Residual), separately calculated for both parts of the begging call. Note that variance components for ID of mean amplitude might be overestimated since the measure was not standardized across individuals.

^a ^Estimates for the effect of hunger are given in change per hour. Number of dF = 294 for all tests. We used 1539 calls from 46 individuals and 27 nests for all tests. Bold typing indicates results that remain significant after correction for multiple testing (*a *= 0.0025, Bonferroni).

Most acoustic call characteristics were affected by the hunger treatment. Mean amplitude, mean entropy and mean pitch goodness increased in both call parts (Table 1). Calls became louder, harsher and the energy distribution became less harmonic. Other call parameters were affected in only one part of the call. Mean frequency and pitch decreased with hunger in the first call part, but were unaffected in the second part (Table 1). Amplitude modulation decreased and frequency modulation increased in the second part (Table 1). The first 'whistle' part became slightly shorter, but the second 'trill-like' part became significantly longer (see duration, Table1). The duration of the second part is strongly correlated to the number of trill elements (LMM: b ± SE = 18.19 ± 0.57 ms/trill, p < 0.0001, N = 31 individuals). An increase of duration in the second part of the call is therefore caused by adding more trill elements.

### Variance in calls in relation to hunger, individuality and nest

In the next step we estimated and compared variation due to the hunger treatment and variation due to individuality. Call parameters important for individual recognition are predicted to be both largely independent of hunger level and highly individually distinct. Mean frequency modulation fits these predictions by showing a very high individuality in both call parts and an independence from hunger level in the first part of the call (variance components, Table 1). Interestingly, most of the other 20 acoustic parameters also showed relatively higher percentages of variation due to individual differences compared to differences in hunger level (variance components, Table 1). Acoustic parameters could not be strictly divided into two categories (i.e. static individual variation and dynamic hunger signal) as predicted. All 20 parameters showed higher variance components for individual effects compared to hunger effects. The effect due to nest of origin (i.e. growing conditions, maternal and genetic effects) was highly variable for different acoustic parameters (ranging from 0 to 37%, Table 1). Of the 20 parameters from both call parts, 11 parameters showed variance components larger than 10% (8 in the first part, 3 in the second part).

### Individuality in calls

Complementary to the variance components for individuals, PIC values gave a second estimate of acoustic individuality. Values for PICs over all hunger levels and variance components for individuals were related (F_1,16 _= 11.60, R^2 ^= 0.42, p = 0.003, N = 18). Six out of 18 acoustic parameters showed PIC values higher than one when PIC was calculated over all hunger states (see additional file 1). The durations of call parts and mean frequency modulation showed PIC values higher than one in both call parts. Additionally, in the first call part mean frequency and mean entropy showed values above one. When calculated for a standardized hunger level (i.e. 105-120 min), 13 out of 18 acoustic parameters showed PIC values higher than one and showed an overall higher individuality than PIC values over all hunger levels (paired T test; t _16 _= 6.75, p < 0.0001, N = 18).

We performed two different sets of DFAs to test for acoustic differences between individuals, i.e. the probability with which a call can be assigned to an individual chick. The results of the first set of DFAs showed that although hunger changes increased, individual chicks can be statistically discriminated with correct assignment rates well above a by-chance correct assignment rate of 2.2%. DFAs using pooled acoustic parameters of both call parts showed higher assignment probabilities (71.3% correct assignment rate) than DFAs that only included call parameters of part 1 (48.8%) or part 2 (45.0%),.

**Figure 1:**
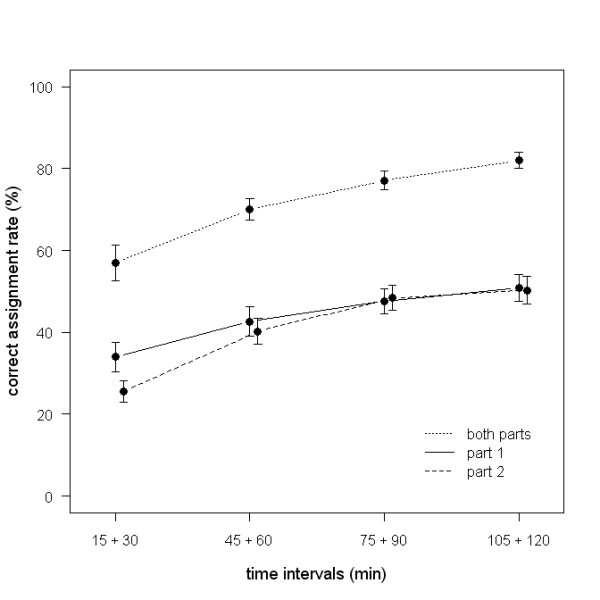
**DFAs on different hunger stages**. Correct assignment rates for DFA on part 1, part 2 and both parts combined on calls from four different time intervals during the treatment (mean ± SE). Note that assignment rates increase with increasing hunger and the assignment rates for both parts together are almost twice as high as for single parts.

In a second set of DFAs, we investigated whether individuality in calls increased with increasing hunger. Individual calls are highly distinctive during all hunger states (for details see Figure 1) and correct call assignment rates increased with increasing hunger (LMM: part 1: b ± SE = 10.2 ± 2.3 percent/h, p < 0.0001; part 2: 15.6 ± 2.1, p < 0.0001; both parts: 16.2 ± 2.2, p < 0.0001; all N = 46; see Figure 1). Calls of hungry nestlings showed higher individuality compared to calls of satiated nestlings. Again, DFAs using pooled acoustic parameters of both call parts showed higher assignment probabilities than DFAs on one part only.

## Discussion

Overall, we experimentally showed that begging calls contain information about a nestling's hunger, and that begging calls remain statistically distinguishable (i.e. show potential for individual recognition) over changes in hunger states. Few acoustic parameters did not change with hunger level and have the potential to act as static recognition cues. However, a strict categorization into static potential identity cues and dynamic, hunger-related components was not possible for most parameters. Several dynamic traits that varied with hunger level likewise showed high individual variation and could therefore potentially function as individual recognition cues. Here, we further develop a hypothesis about how individual recognition processes can incorporate dynamic hunger-related information, and the potential consequences for the correct assessment of a chick's hunger level.

### Call complexity

The structure of Jackson's golden-backed weaver nestlings' calls is highly complex and can be classified into two distinct parts: a 'whistle-like' first part and a 'trill-like' second part. This two-parted structure emerges when nestlings are around 10 days of age and remains this complex until at least 36 days of age (own unpublished data). A similar change in complexity of contact or begging calls close to fledging has been demonstrated in the chaffinch (*Fringilla coelebs*) [26] and in the bank swallow (*Riparia riparia*) [27,28]. In these two species, the call increases in amplitude, becomes longer and starts showing a complex time-frequency pattern close to fledging. This increase in acoustic complexity prior to fledging is thought to facilitate recognition by allowing the parents to learn the individual signature before the nestling leaves the nest [28]. Our results support this hypothesis. Although DFAs using differing numbers of variables are not directly comparable, the higher assignment rate of the DFAs using acoustic parameters of both call parts demonstrates that a composite call, i.e. increased complexity within a signal, provides enhanced discrimination compared to each call part alone (Figure 1).

### Acoustic variability within and between individuals

Most acoustic parameters in both call parts changed in response to an increase in a nestling's hunger state. 12 out of 20 parameters were strongly related to a nestling's hunger state. As predicted, amplitude increased with hunger [e.g. [2,21]]. Changes in amplitude, however, may partly be explained by decreased distances to the microphone caused by posture changes of the nestling. The second part of the call was extended by an increase in the number of trill elements. This result confirms findings on barn swallows (*Hirundo rustica*) [24] and tree swallows (*Tachycineta bicolour*) [3], showing that call duration conveys information about nestlings' hunger. These results indicate that acoustic parameters could be used by the parents to assess a nestling's need based on reliable changes with hunger. However, experimental evidence, i.e. playback experiments testing specific acoustic parameters, is necessary to test whether parents perceive and use hunger related variation as a signal of need.

Compared to the number of call parameters that were affected by a nestling's hunger, only a few parameters remained unaffected and static over all hunger levels. As found for other species, frequency modulation contained high individual variation, especially in the first part of the call. In addition, frequency modulation was not influenced by changes in hunger (Table 1 and additional file 2). This suggests that frequency modulation might act as an important part of an individual signature system in Jackson's golden-backed weavers. Additionally, amplitude modulation and variance in entropy were largely unaffected by hunger in the first part of the call, while mean frequency and variance in entropy were unaffected by hunger in the second call part. However, individual recognition is most likely not based on one single component showing high individuality, but on a multitude of components and their complex interactions [1,14,29]. The integration of multiple components may translate into fitness benefits due to enhanced perception of signal information (redundant signal hypothesis [30]) or due to the possibility to perceive multiple, partly independent information (multiple message hypothesis [30]). According to the variance components for individuality and the PIC values, nestling calls show potential for individual recognition in several acoustic parameters. This can enable parents to learn their nestlings' individual signature close before fledging, allowing them to relocate their offspring after leaving the nest. However, individual recognition experiments on this species are still missing. The importance of call components for potential individual recognition processes remains unknown, and needs to be addressed in future playback experiments testing the receiver's perception of differences in specific call parameters.

Acoustic individuality can also be shown when taking a multivariate approach. The DFAs over all hunger levels demonstrate that even though begging calls change with hunger, individuals can still be statistically discriminated based on acoustic parameters. Acoustic individuality is therefore maintained over changing hunger. The correct assignment rates of the DFAs increased with hunger (Figure 1). This finding could be expected, given that individual recognition is more important in young in high state of need. Alternatively, this finding might be due to motivational differences over the different hunger levels. Very hungry, fully motivated nestlings might produce well-stereotyped calls at maximum physiological effort, resulting in an increased acoustic variance between individuals. In contrast satiated nestlings may differ in their motivation from call to call and therefore produce less stereotyped calls which show a larger overlap between individuals.

Interestingly, most acoustic parameters showed a combination of high individuality and reliable hunger signalling. A strict categorization into static or dynamic parameters is therefore not valid. Instead, candidate cues for individual recognition also incorporate dynamic, hunger-related variation, thereby potentially signalling the nestling's hunger to the parents. For example, the duration of part 2 of the call shows clear differences between individuals (i.e. high individuality) and, simultaneously, the duration increases with hunger (Table 1 and additional file 2). These signal properties raise an interesting theoretical concept about a combination of identity and condition signalling. The absolute duration of part 2 is not a reliable indicator of a chick's hunger by itself. However, when duration of part 2 is perceived on a relative scale (i.e. compared within an individual's acoustic spectrum), the perceiver can obtain a highly accurate estimate of a chick's state of need. One option is that receivers may use a general 'rule of thumb' in which they compare among several begging characteristics or relate begging characteristics to begging posture in order to assess a chick's state of need. Alternatively, receivers compare the signal with an 'individual-referenced' template of the acoustic spectrum of the sender, and thereby perceive more information about the sender's condition. In other words, by knowing the identity of the sender and being familiar with the sender's acoustic range, the receiver could improve perception of signals. While the acoustic range of the signaller may change with age, body condition or status, repeated interactions in parent-offspring communication may ensure that parents stay familiar with the acoustic spectrum of individual chicks.

Perceiving more precise acoustic information about an individual through familiarity with its acoustic signal spectrum is presumably a common, though not yet well-investigated, phenomenon. To our knowledge, very few studies on non-human animals have investigated the effect of familiarity on perceiving individuals' acoustic signals. Studies on vocalizations of great tits (*Parus major*) [31,32] and western meadowlarks (*Sturnella neglecta*) [33] found that familiarity with the songs of an individual improves ranging estimates by the receiver. McGregor and Krebs [31] demonstrated in great tits that territory holders react differently to degraded and not-degraded songs only when they are familiar with the opponent's song. They suggested that the receiver compares a familiar song to a learned template in order to estimate the degradation of the song, and therefore cannot judge the degradation of unfamiliar songs. A very similar ranging effect has been demonstrated in humans by the same authors [34]. Further studies in humans suggest that being familiar with a specific voice increases the ability to recognize words that are overlaid with noise [35-37]. Those examples indicate that perceiving certain information requires familiarity with the signal variability of an individual. These studies highlight that individual-referenced signalling could be a widely used communication component in social communication systems, which allow learning of acoustic signal templates through repeated interactions.

## Conclusions

Here, we used a signaller's perspective to experimentally disentangle the fine-scaled variation in multi-component begging calls of Jackson's golden-backed weaver chicks. However, to fully understand signalling systems one has to adopt a signaller's and a receiver's perspective. Thus, playback experiments are clearly needed to demonstrate whether receivers assess signal value or single call components and if those signals are assessed on an absolute scale or referenced to the acoustic spectrum of a signaller.

## Methods

### Field study

**Figure 2:**
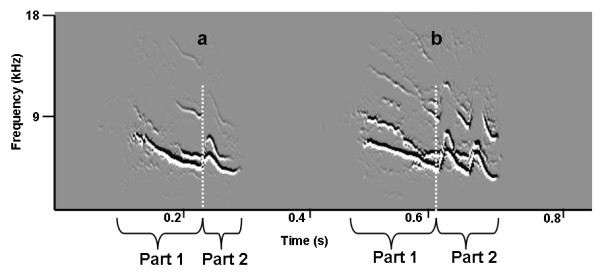
**Effect of hunger on calls**. Spectrograms of representative calls of a 13 days old chick (A) 15 minutes and (B) 120 minutes after food deprivation. Note the two distinct parts of the call and the differing number of trills in the second part of the calls (i.e. one trill in (A) and two trills in (B). Spectrograms are drawn using SAP.

**Figure 3:**
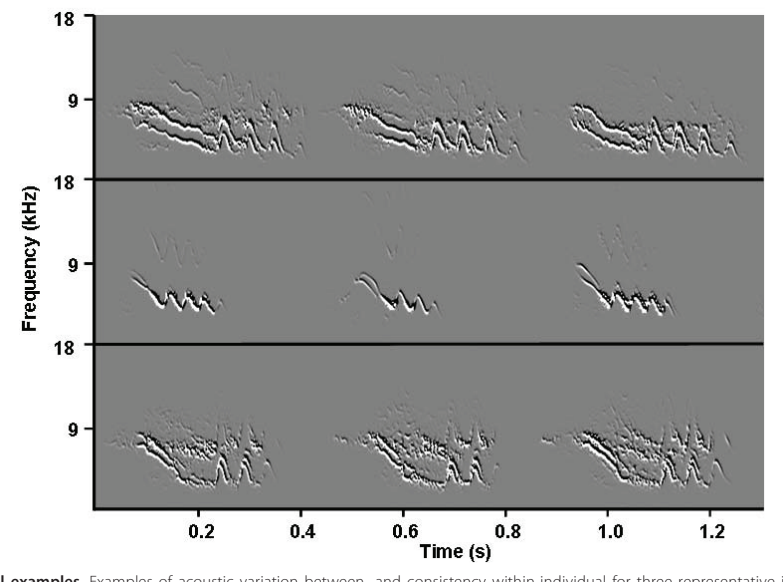
**Call examples**. Examples of acoustic variation between- and consistency within-individual for three representative individuals. All shown calls are from individuals after 120 minutes of food deprivation. Spectrograms are done using SAP.

This study was conducted on the western shores of Lake Baringo/Kenya (N 0°36"40"; E 36°1"20") in the East African Rift Valley. The Jackson's golden-backed weaver is a colonial breeder with colony sizes reaching 200 nests. Breeding starts at the beginning of the rainy season (April to September), but the precise onset of breeding depends on the occurrence of the first rains and therefore varies between years. Males are polygynous and build up to five nests. Females choose nests and lay two to three eggs. The incubation period is about 14 days and nestlings hatch asynchronously because incubation starts with the first egg. In our colony, nestlings fledged 17 ± 2 days after hatching. Most nestlings within the colony fledged very synchronized (i.e. within around a week) [own unpublished data, 25]. Like other weaver species, fledglings are fed for about 2-3 weeks, in most cases exclusively by the mother [25]. Begging calls of *P. jacksoni *change during ontogeny from a simple to a complex call that shows two distinctive parts (own unpublished data). The first part of the call is whistle-like, descending in frequency, while the second call part consists of repeated elements that show an upside down U-shaped pattern in spectrograms and sounds like a short trill (Figures 2 and 3). All analyses were done separately for each call part.

We monitored nests during the breeding seasons of 2007 and 2008. Nests were checked daily during laying onset, hatching and fledging dates. Nestlings were marked individually by plucking down feathers on the head on the day of hatching and with individually numbered alloy rings on day 8. To investigate the effect of hunger-related variation on behavioural and acoustic begging characteristics, we performed a food deprivation experiment with 49 nestlings from 29 nests (1-2 nestlings/nest). Food deprivation experiments were performed shortly before fledging (2007: N = 17 birds, 13 ± 1 days old; 2008: N = 32 birds, 12 ± 0 days old). Three of those 49 nestlings did not accept manual feeding and were excluded from further analysis. Prior to the experiment, all chicks were weighed with an electronic balance (CM 150, Kern, Balingen-Frommern, Germany) to the nearest 0.1 g (mean ± sd, 16.7 ± 2.6, N = 49) and tarsus length was measured with slide callipers to the nearest 0.1 mm (mean ± sd, 19.4 ± 1.1, N = 49). After the experiment, a small blood sample was collected for molecular sexing (23 males, 23 females) [38]. We were not able to collect enough blood from three nestlings for sexing.

### Food deprivation and recording of begging calls

In the food deprivation experiment, nestlings were temporarily removed from the natal nest and moved to an artificial nest cup, made from a plastic box (6 cm in diameter) and a cloth lining. The experimental setup was located 50-150 m from the colony at the lake shore. We placed one nestling at a time in its own artificial nest cup at a fixed position inside the experimental box (card board, 40 × 40 × 20 cm, width × depth × height). To decrease echoes and ambient noise, the box was sound-shielded on the inside with acoustic foam (N04HG, http://schaumstoff.com, Bochum, Germany). Prior to the experiment, nestlings were fed rearing food pellets (NutriBird C15, Versele-Lage GmbH, Wesel, Germany) until they did not accept any more food. The procedure ensured the standardization of hunger levels at the start of the experiment. During this satiation process hungry nestlings readily responded to the stimulus (see below) with begging behaviour, but became gradually less responsive with increasing satiation. After satiation, begging behaviour was induced and recorded every 15 minutes for the next two hours, starting 15 minutes after satiation [for similar protocol see: 20]. Begging behaviour was induced in a standardized way by gently jolting the nest cup, simultaneously producing three consecutive soft broadband, noisy sounds with the lips and feeding one food pellet (about 0.05 g). Own preliminary studies had shown that feeding a small food pellet elicits the most repeatable begging response, while chicks were still getting hungrier. Most importantly, our treatment ensures that all chicks were fed the same number of food pellets over the experimental time period.

A microphone (C2, Behringer, Willich, Germany) was placed in a fixed position 8 cm directly above the artificial nest cup and the fledglings' calls were recorded at a sampling rate of 44.1 kHz and 16 bit amplitude resolution onto a solid state recorder (Microtrack II, M-Audio, Irwindale, USA). In order to confirm that the treatment resulted in an increase in hunger, we filmed the nestling during the experiment to quantify changes in begging posture (JVC GZ-MG77, Yokohama, Japan). Maximum begging posture was categorized into five states adapted from previous studies [17,39,40]: 1) no reaction; 2) opening bill, but refusing to feed; 3) acoustic begging, little wing flap, neck not stretched; 4) acoustic begging with wing flap and/or neck stretched; 5) strong acoustic begging, neck stretched all the way, standing up and flapping wings. After the experiment, the chicks were fed until satiation and placed back into their original nests. All nestlings were accepted once they were put back into the nest and no premature fledging was observed.

### Sound analysis

Hunger-related variation in acoustic begging behaviour was measured using the nestling's quantitative and qualitative response. As a quantitative variable we measured call rate by counting the number of begging calls in the 10 seconds following the first begging call emitted in response to a stimulus.

The qualitative response of nestlings was measured as the maximum intensification of a nestling's acoustic begging, estimated as the calls with the highest amplitude [3]. When nestlings respond to a stimulus, their response pattern shows often a clear pattern with high-motivation calls shortly after the stimulus and a continuous decrease in motivation thereafter (see additional file 3). Therefore, taking random calls for the analysis of a nestling's hunger-related changes in call characteristics may not capture the biologically important information. The hunger-related qualitative changes in begging call characteristics were analysed by manually selecting the five calls with the highest response to the stimulus (i.e. calls with the highest amplitude). Using a sub-sample of 23 nestlings for which we randomly selected calls we confirmed that our call selection lead to similar estimation of call parameters (see additional file 4).

Begging calls were analysed using the computer program Sound Analysis Pro 2.063 (SAP) [for details see [41]]. Compared to other methods that use visually assessed measurements from spectrograms, SAP uses complex algorithms to calculate values for each millisecond of the call and provides means and variances of those values. The begging calls of *P. jacksoni *nestlings consist of two distinct parts (Figure 2). For analysis we derived 10 acoustic parameters for each call part separately: 1) duration of call part (in ms); 2) mean amplitude (in dB); 3) variance of amplitude modulation (in 1/ms); 4) mean frequency (in Hz); 5) mean frequency modulation (in Hz); 6) variance of frequency modulation (in Hz); 7) mean entropy; 8) variance in entropy; 9) mean pitch (in Hz) and 10) mean pitch goodness.

The amplitude measure was not standardized between recordings of different nestlings but was consistent within nestlings. We therefore used amplitude only to measure within-individual changes. All other acoustic measures are independent of the absolute amplitude and are therefore unbiased by amplitude differences between recordings. Frequency modulation is an estimate of changes in frequency over time with high values meaning high frequency changes over time and vice-versa. Amplitude modulation is the change in amplitude over time; high values represent high changes in amplitude. Mean frequency provides a smooth estimate of the frequency with the highest power. It is calculated as mean frequency, weighted by amplitude, and therefore does not 'stick' to any frequency trace within the spectrogram. Entropy is a measure of how noisy a sound is; pure tones show low entropy, while broadband sounds show high entropy. Pitch as measured by SAP is an estimate of the fundamental frequency, based on how harmonic a sound is. For tonal sounds (e.g. a whistle) pitch is estimated as mean frequency; for harmonic sounds pitch is the fundamental frequency. The measure is weighted by pitch goodness, giving harmonic sounds more weight than tonal sounds to get a more robust measure of fundamental frequency. Pitch goodness measures the harmonic richness of a sound; low pitch goodness indicates a sound with strong harmonics while high pitch goodness indicates a pure tone without harmonics. Variances of acoustic parameters are a measure of changes over time. A high variance means high changes over time; low variance indicates little changes over time [for details see [41]].

The start and the end point of the overall call was automatically assessed in SAP by an amplitude-threshold of 25 dB and an entropy-threshold of -1.3 [for details see [41]]. These values provided the best separation of calls from background noise. Calls were then manually separated into two parts by one observer. The cut-off was defined as the lowest point of the loudest frequency band, just before the repeated trill part (Figure 2).

### Statistical analysis

#### General statistical analysis

All statistical analyses were performed with R2.8.1 [42]. Variance of entropy from part 1 and 2 and variance of amplitude modulation from part 2 were log-transformed to approach normality. The effect of hunger on begging behaviour and acoustic call parameters was estimated in linear mixed effect models (LMM) [43]. The potential for individual identity coding (PIC) was assessed for every acoustic parameter [44]. Finally we used a discriminant function analysis (DFA) to quantify discrimination potential for individuals in relation to hunger [package MASS, [45]].

#### Hunger effects on begging

The effect of hunger on begging posture, number of calls and acoustic call parameters was estimated by including year as a categorical fixed factor, hunger state (ranging from 15 - 120 minutes in steps of 15 min) as a continuous covariate and individual and nest identity as random factors. We extracted variance components from LMMs on the effect of hunger on acoustic parameters to apportion the observed variance to hunger state, nest identity and individual identity. In those LMMs, we used year as a fixed factor and hunger state, nest and individual as random effects. Sex, body mass, tarsus length and hatching order were initially included as covariates into the mixed-effects models for effects of hunger on begging posture, number of calls and acoustic parameters. Out of 88 tests, only 6 were borderline significant (range: p = 0.0104-0.0472), but became not significant after adjusting the significance level to *a *= 0.0006 using the Bonferroni method [46]. Those parameters were excluded from the final models, thereby simplifying the models. The fact that neither body mass nor tarsus length had an effect on acoustic parameters is most likely due to limited variance in those variables, even across sexes. Year was included in all models to account for age (chicks were recorded at 13 days of age in 2007 and 12 days of age in 2008) and season effects. The standard model diagnostics of non-normal errors, non-constant error variance and the presence of outliers were performed on each of the final models according to Fox [47].

#### Potential for individual coding

PIC is a measure of the ratio of inter-individual variation in comparison to intra-individual variation. To describe the intra- and inter-individual variations of each variable, we used the coefficient of variation (CV). For each variable we calculated CVi (intra-individual CV) and CVb (inter-individual CV) according to the formula: *CV *= 100 × (*SD*/*X*), where *SD *is the sample standard deviation and *X *is the sample mean [44]. PIC is the ratio of CVb divided by the mean of CVi of all individuals. For a given variable, a PIC value greater than one suggests that this variable may be used for individual recognition since its intra-individual variability is smaller than its inter-individual variability. PICs were calculated for parameters both over all hunger levels pooled and for the maximum hunger level (i.e. at 120 min). PICs were not calculated for mean amplitude since amplitude was not standardized across individuals.

#### Discriminant function analyses

We performed two sets of DFAs to statistically investigate individuality. In the first DFA, we investigated whether calls stay individually distinctive over all hunger states. This analysis is performed without information about a chick's hunger state. A high chick assignment rate would demonstrate that a chick's voice remains distinctive independent of its hunger state. In a second set of DFAs we tested how individual discrimination is affected by changes in hunger. Here, we performed separate DFA's on four time intervals of food deprivation: i) 15 and 30; ii) 45 and 60; iii) 75 and 90; iv) 105 and 120 minutes. Creating time intervals, i.e. lumping 2 time points, was necessary because several individuals only called once during a given time point, but at least 2 calls per individual are required to calculate a cross-validated DFA (see below). Following the DFAs, we performed a linear mixed-effects model with individual as random factor and average assignment rate (four levels per individual) as continuous variable to investigate changes in individual discrimination over hunger states. For each set we conducted DFAs on both parts of the calls separately and on both parts combined. For all DFAs we used 9 call parameters (all but mean amplitude) per call part, and 18 call parameters for DFAs on both parts combined respectively. All DFAs were done using a cross-validated (leave-one-out) procedure, which fits the left out call into a multidimensional signal space computed from all calls except the one which was left out. The left-out call was then assigned with a certain probability to each individual based on the Mahalanobis distances from each call to the centroid of each individual [package: MASS, [45]].

### Ethical note

This study has been approved by the Kenyan Ministry of Science and Technology and the National Museums of Kenya (permit number: MOST 13/001/38C251). We did not encounter any problem with nestling survival during or following the experiments and fledging rate was comparable to untreated nests.

## Authors' contributions

HR participated in study design, performed the experiments and the statistical analysis and drafted the manuscript. AJ participated in study design, performing experiments and drafting the manuscript. Both authors read and approved the manuscript.

## Supplementary Material

Additional file 1 PIC values for all acoustic parameters both over all hunger stages and maximum hunger stage. Click here for file

Additional file 2 Four example plots of the effect of hunger on acoustic parameters. Click here for file

Additional file 3 Response pattern of nestlings Click here for file

Additional file 4 Comparison between maximum response calls and randomly chosen calls Click here for file
