# Supplementary material for: The effect of hunger on the acoustic individuality in begging calls of a colonially breeding weaver bird
Source: BMC Ecol. 2011 Jan 26;11:3. doi: 10.1186/1472-6785-11-3 (PMC3038888; doi:10.1186/1472-6785-11-3)
Supplement: Additional file 1 — PIC values for all acoustic parameters both over all hunger stages and maximum hunger stage. [file 1472-6785-11-3-S1.PDF]

**Additional file 1:**

PICs for acoustic parameters over all hunger levels and over standardized hunger after 105-120 minutes. Bold typing indicates parameters that show potential for individual recognition.

| | Acoustic parameters | | PIC over all hunger levels | | | | PIC over 105-102 minutes of food deprivation | | | |
|---|---|---|---|---|---|---|---|---|---|---|
| | | | mean ± sd | meanCVi | CVb | PIC | mean ± sd | meanCVi | CVb | PIC |
| Part1 | Duration (ms) | | 118.37 ± 22.31 | 13.71 | 18.85 | **1.37** | 118.03 ± 22.95 | 10.32 | 19.56 | **1.90** |
| | Amplitude modulation (1/ms) | variance | (6.15 ± 0.92) x$10^{-3}$ | 20.17 | 14.95 | 0.74 | (6.25 ± 0.95) x$10^{-3}$ | 18.43 | 15.28 | 0.83 |
| | Frequency (Hz) | mean | 6767.72 ± 572.29 | 7.65 | 8.46 | **1.11** | 6652.35 ± 588.52 | 6.61 | 8.90 | **1.35** |
| | Frequency modulation | mean | 23.72 ± 6.55 | 20.50 | 27.62 | **1.35** | 23.87 ± 6.04 | 16.67 | 25.43 | **1.53** |
| | Frequency modulation | variance | 463.46 ± 90.47 | 20.58 | 19.52 | 0.95 | 476.87 ± 85.67 | 18.39 | 18.06 | 0.98 |
| | Entropy | (log) mean | -3.50 ± 0.48 | -11.33 | -13.65 | **1.20** | -3.39 ± 0.47 | -9.81 | -14.06 | **1.43** |
| | Entropy | variance | 0.47 ± 0.12 | 34.80 | 26.31 | 0.76 | 0.47 ± 0.15 | 33.72 | 31.70 | 0.94 |
| | Pitch (Hz) | mean | 3137.02 ± 607.72 | 21.96 | 19.37 | 0.88 | 3013.80 ± 623.02 | 18.55 | 20.79 | **1.12** |
| | Pitch goodness | mean | 203.12 ± 46.02 | 23.05 | 22.66 | 0.98 | 216.72 ± 53.62 | 20.05 | 24.88 | **1.24** |
| Part2 | Duration (ms) | | 60.69 ± 16.21 | 23.46 | 26.71 | **1.14** | 70.59 ± 19.14 | 13.61 | 27.26 | **2.00** |
| | Amplitude modulation (1/ms) | (log) variance | (1.55 ± 0.57) x$10^{-3}$ | 69.37 | 36.44 | 0.53 | (1.45 ± 0.64) x$10^{-3}$ | 56.82 | 43.97 | 0.77 |
| | Frequency (Hz) | mean | 5634.74 ± 484.00 | 9.24 | 8.59 | 0.93 | 5635.27 ± 558.43 | 8.41 | 9.96 | **1.18** |
| | Frequency modulation | mean | 40.56 ± 6.97 | 13.41 | 17.18 | **1.28** | 41.47 ± 7.10 | 10.78 | 17.23 | **1.60** |
| | Frequency modulation | variance | 599.90 ± 69.25 | 16.91 | 11.54 | 0.68 | 614.84 ± 78.79 | 14.42 | 12.89 | 0.89 |
| | Entropy | (log) mean | -3.46 ± 0.47 | -13.80 | -13.50 | 0.98 | -3.30 ± 0.51 | -11.27 | -15.59 | **1.38** |
| | Entropy | variance | 0.43 ± 0.11 | 35.02 | 25.64 | 0.73 | 0.42 ± 0.12 | 27.68 | 29.51 | **1.07** |
| | Pitch (Hz) | mean | 3273.24 ± 624.57 | 21.40 | 19.08 | 0.89 | 3229.33 ± 664.07 | 18.00 | 20.68 | **1.15** |
| | Pitch goodness | mean | 240.14 ± 58.76 | 25.01 | 24.47 | 0.98 | 264.08 ± 63.04 | 19.66 | 24.00 | **1.22** |
