# Supplementary material for: The effect of hunger on the acoustic individuality in begging calls of a colonially breeding weaver bird
Source: BMC Ecol. 2011 Jan 26;11:3. doi: 10.1186/1472-6785-11-3 (PMC3038888; doi:10.1186/1472-6785-11-3)

Additional file 2:

Four examples for different trait properties: a) ID trait with low intra- but high inter-individual variation; b) hunger and ID trait with both high intra- and inter-individual variation; c) hunger trait with high intra- but low inter-individual variation; d) neither hunger nor ID trait. For easier interpretation values for each individual and time interval are averaged and connected by lines to show individual changes with hunger.

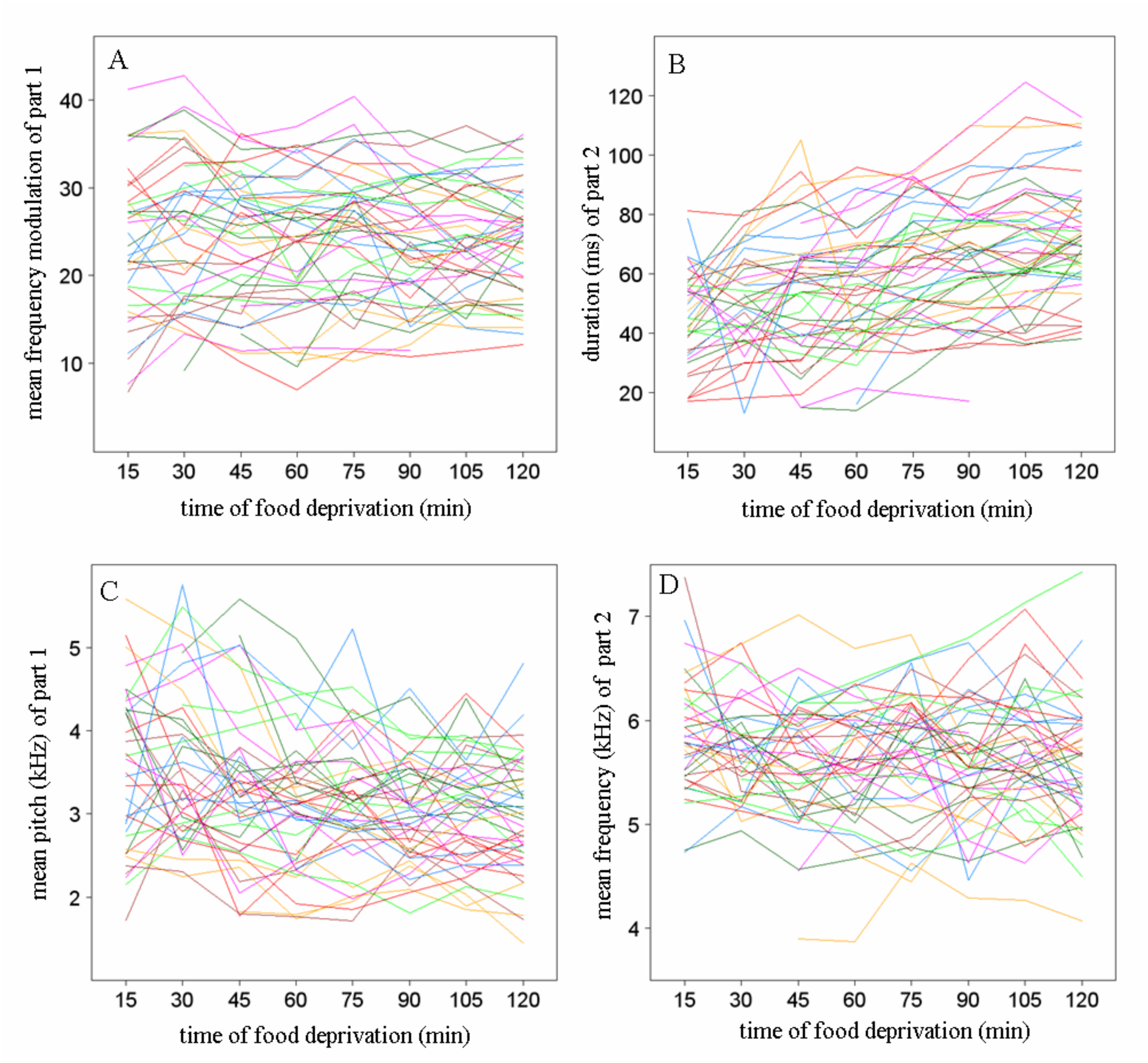

Supplement: Additional file 2 — Four example plots of the effect of hunger on acoustic parameters. [file 1472-6785-11-3-S2.PDF]
