# Supplementary material for: The effect of hunger on the acoustic individuality in begging calls of a colonially breeding weaver bird
Source: BMC Ecol. 2011 Jan 26;11:3. doi: 10.1186/1472-6785-11-3 (PMC3038888; doi:10.1186/1472-6785-11-3)

Additional file 3:

Representative response pattern of nestlings to feeding stimulus. Shown is the response during 10 seconds following a stimulus (first very short and broadband sound) after 60 minutes of food depravation. Call ‘intensity’ decreases after an initially rigorous response. To measure the need of nestlings, we were mostly interested in the maximum response.

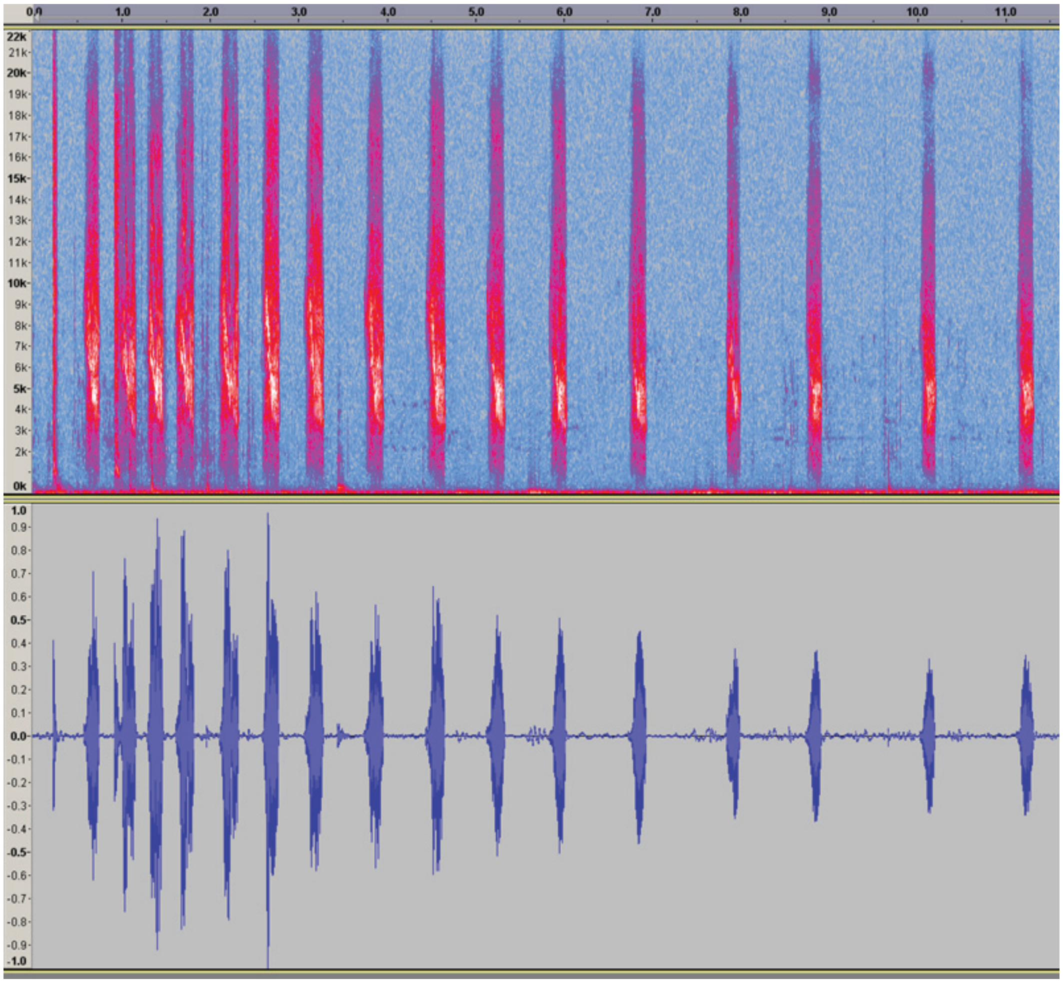

Supplement: Additional file 3 — Response pattern of nestlings [file 1472-6785-11-3-S3.PDF]
