# Supplementary material for: The effect of hunger on the acoustic individuality in begging calls of a colonially breeding weaver bird
Source: BMC Ecol. 2011 Jan 26;11:3. doi: 10.1186/1472-6785-11-3 (PMC3038888; doi:10.1186/1472-6785-11-3)
Supplement: Additional file 4 — Comparison between maximum response calls and randomly chosen calls [file 1472-6785-11-3-S4.PDF]

Additional file 4:

Table 1:

Paired T-test for differences between highest response and randomly sampled calls of **satiated (i.e. 15 and 30 minutes)** nestlings. Significance level has been adjusted for multiple testing to α=0.0025 after Bonferroni, significant results are indicated in bold typing.

	Acoustic parameter		Highest response Mean ± SD	Random sample Mean ± SD	Difference t	p-value
Part 1	Duration (ms)		117.3 ± 28.0	122.5 ± 37.8	-1.95	0.0651
	Amplitude (dB)	mean	42.1 ± 3.5	41.2 ± 3.7	4.19	**0.0004**
	Amplitude modulation (1/ms)	variance	0.0063 ± 0.0009	0.0062 ± 0.0008	0.57	0.5774
	Frequency (Hz)	mean	7076.5 ± 544.0	7074.9 ± 508.4	0.06	0.9553
	Frequency modulation	mean	27.2 ± 7.1	26.8 ± 6.9	1.83	0.0810
	Frequency modulation	variance	489.0 ± 100.4	483.6 ± 89.3	0.91	0.3716
	Entropy	mean	-3.41 ± 0.35	-3.44 ± 0.39	1.37	0.1856
	Entropy	variance	0.62 ± 0.22	0.59 ± 0.20	2.30	0.0316
	Pitch (Hz)	mean	3407.8 ± 714.1	3428.3 ± 672.9	-0.50	0.6222
	Pitch goodness	mean	191.3 ± 34.1	183.9 ± 31.6	1.97	0.0616
Part 2	Duration (ms)		51.1 ± 14.0	49.3 ± 16.5	1.60	0.1250
	Amplitude (dB)	mean	40.0 ± 4.5	37.8 ± 4.7	4.58	**0.0002**
	Amplitude modulation (1/ms)	variance	0.0017 ± 0.0006	0.0017 ± 0.0007	0.04	0.9665
	Frequency (Hz)	mean	5747.2 ± 448.1	5673.7 ± 431.1	1.85	0.0792
	Frequency modulation	mean	41.6 ± 4.7	42.8 ± 5.4	-1.30	0.2071
	Frequency modulation	variance	572.3 ± 60.3	557.4 ± 80.6	1.13	0.2724
	Entropy	mean	-3.47 ± 0.46	-3.45 ± 0.40	-0.58	0.5650
	Entropy	variance	0.52 ± 0.17	0.51 ± 0.18	0.18	0.8604
	Pitch (Hz)	mean	3305.9 ± 780.8	3308.3 ± 749.5	-0.05	0.9603
	Pitch goodness	mean	216.2 ± 53.7	206.5 ± 42.4	2.39	0.0264

Table 2:

Paired T-test for differences between highest response and randomly sampled calls of **hungry (i.e. 105 and 120 minutes)** nestlings. Significance level has been adjusted for multiple testing to α=0.0025 after Bonferroni, significant results are indicated in bold typing.

	Acoustic parameter		Highest response Mean ± SD	Random sample Mean ± SD	Difference t	p-value
Part 1	Duration (ms)		109.1 ± 20.3	104.0 ± 17.6	2.27	0.0334
	Amplitude (dB)	mean	46.0 ± 3.6	45.3 ± 3.8	2.37	0.0271
	Amplitude modulation (1/ms)	variance	0.0064 ± 0.0010	0.0066 ± 0.0011	-1.49	0.1497
	Frequency (Hz)	mean	6659.9 ± 457.0	6389.9 ± 559.4	3.24	0.0038
	Frequency modulation	mean	26.4 ± 5.3	25.7 ± 5.5	1.53	0.1413
	Frequency modulation	variance	503.0 ± 69.4	495.4 ± 78.0	1.07	0.2950
	Entropy	mean	-3.22 ± 0.47	-3.26 ± 0.46	-0.10	0.9202
	Entropy	variance	0.59 ± 0.27	0.59 ± 0.29	-0.03	0.9789
	Pitch (Hz)	mean	2970.7 ± 549.8	2913.4 ± 619.7	0.90	0.3780
	Pitch goodness	mean	226.6 ± 52.3	227.1 ± 49.6	-0.09	0.9321
Part 2	Duration (ms)		75.0 ± 22.0	73.7 ± 20.5	1.09	0.2877
	Amplitude (dB)	mean	46.3 ± 4.8	45.4 ± 5.1	3.83	**0.0009**
	Amplitude modulation (1/ms)	variance	0.0013 ± 0.0003	0.0015 ± 0.0007	-1.51	0.1450
	Frequency (Hz)	mean	5638.2 ± 464.8	5414.6 ± 540.3	2.85	0.0093
	Frequency modulation	mean	43.3 ± 5.4	42.7 ± 4.9	2.08	0.0491
	Frequency modulation	variance	595.8 ± 64.8	581.4 ± 64.0	2.05	0.0526
	Entropy	mean	-3.19 ± 0.57	-3.30 ± 0.57	4.12	**0.0005**
	Entropy	variance	0.506 ± 0.153	0.509 ± 0.170	-0.17	0.8692
	Pitch (Hz)	mean	3248.5 ± 563.9	3178.1 ± 671.6	0.95	0.3549
	Pitch goodness	mean	258.2 ± 56.2	255.2 ± 59.8	0.56	0.5793
